# Passive Sensor Integration for Vehicle Self-Localization in Urban Traffic Environment †

**DOI:** 10.3390/s151229795

**Published:** 2015-12-03

**Authors:** Yanlei Gu, Li-Ta Hsu, Shunsuke Kamijo

**Affiliations:** Institute of Industrial Science, The University of Tokyo, 4-6-1 Komaba, Meguro-ku, Tokyo 153-8505, Japan; qmohsu@kmj.iis.u-tokyo.ac.jp (L.-T.H.); kamijo@iis.u-tokyo.ac.jp (S.K.)

**Keywords:** vehicle self-localization, sensor integration, 3D map, GNSS, inertial sensor, vision, lane detection, particle filter

## Abstract

This research proposes an accurate vehicular positioning system which can achieve lane-level performance in urban canyons. Multiple passive sensors, which include Global Navigation Satellite System (GNSS) receivers, onboard cameras and inertial sensors, are integrated in the proposed system. As the main source for the localization, the GNSS technique suffers from Non-Line-Of-Sight (NLOS) propagation and multipath effects in urban canyons. This paper proposes to employ a novel GNSS positioning technique in the integration. The employed GNSS technique reduces the multipath and NLOS effects by using the 3D building map. In addition, the inertial sensor can describe the vehicle motion, but has a drift problem as time increases. This paper develops vision-based lane detection, which is firstly used for controlling the drift of the inertial sensor. Moreover, the lane keeping and changing behaviors are extracted from the lane detection function, and further reduce the lateral positioning error in the proposed localization system. We evaluate the integrated localization system in the challenging city urban scenario. The experiments demonstrate the proposed method has sub-meter accuracy with respect to mean positioning error.

## 1. Introduction

Vehicle self-localization in urban environment is a challenging but significant topic for autonomous driving and driving assistance. Both motion planning and vehicle cooperation need accurate localization. Localization methods can be categorized into two types based on the sensors used: passive sensor-based and active sensor-based. Passive sensors such as Global Navigation Satellite System (GNSS) receivers and vision sensors collect signals, radiation, or light from the environment, while the active sensors have transmitters, which can send out light waves, electrons or signals. The reflection bounced off the target will be collected by the active sensor. Velodyne, a type of Light Detection and Ranging (LIDAR) sensor is a representative active sensor for vehicle self-localization [1,2,3,4,5,6,7]. Active sensor-based localization has a weakness from a practical point of view—the price of these sensors is currently very high. Although we expect that the cost of these sensors will be reduced in the near future, they suffer from another critical problem, which is the high energy consumption. Therefore, it is necessary to consider low-cost solutions such as GNSS receivers, cameras and inertial sensors—*i.e*., passive sensors—as an alternative or compensation.

Global Positioning System (GPS) is the main information source in vehicle navigation, which was indicated from the famous Defense Advanced Research Projects Agency (DARPA) GRAND CHALLENGE [8]. Under ideal conditions, namely eight GPS satellites or more in the open sky field, the error standard deviation of the differential mode GPS positioning is approximate 0.3 m [9]. However, vehicles often run in urban areas, in which GNSS positioning performance is severely degraded due to Non-Line-Of-Sight (NLOS) and multipath effects. These effects may lead to a 100 m positioning error in the city urban environment [10]. Various GNSS technologies were proposed to mitigate the NLOS and multipath effects [11]. Recently, building information was considered to analyze the effects of NLOS and multipath. Marais *et al.*, proposed to exclude the satellites located in non-sky regions for localization using the omnidirectional fisheye camera [12]. In addition, 3D building model, shadow mapping, and omnidirectional infrared (IR) cameras were employed for excluding the unhealthy signals and mitigating the effects of NLOS and multipath [13,14,15]. However, the exclusion of satellites will cause the distortion of Horizontal Dilution of Precision (HDOP). Another famous urban positioning method is shadow matching. This method evaluates the satellite visibility using city building models, and compares the predicted satellite visibility with the visibility measurement to reduce the positioning error [16,17,18]. In order to mitigate the NLOS and multipath effects while reducing the HDOP distortion, we proposed a candidate distribution based positioning method using a 3D building map (3D-GNSS) [19,20,21]. 3D-GNSS takes the advantage of 3D building map to rectify the pseudorange error caused by NLOS and multiple effects. The developed 3D-GNSS has been evaluated for pedestrian applications. The result demonstrated 3D-GNSS can obtain high positioning accuracy in urban canyons.

Following the idea of the integration of GPS and inertial navigation systems [22,23,24,25,26], we integrated inertial sensors and 3D-GNSS for vehicle applications as well [27,28]. The evaluation result indicated the inertial sensor can smooth the positioning trajectory, however, the combination of 3D-GNSS and inertial sensors still cannot satisfy the sub-meter accuracy requirement of autonomous driving [29,30]. Moreover, the inertial sensor can output accurate heading direction information during a short time period, but the drift will become obvious as time increases.

In addition to the inertial sensor and GNSS positioning, sensing techniques were also widely used for positioning. The most famous one is Simultaneous Localization and Mapping (SLAM). SLAM is considered as a problem of constructing or updating a map while at the same time tracking the vehicle position in the map. Optical sensors used may be a Velodyne Laser Scanner [2,4,31], camera [32,33,34] or the fusion of both [35]. However, SLAM may display error accumulation problems [4], because the localization and mapping are conducted simultaneously. Therefore, the two-step method which firstly constructs an accurate map off-line, and then matches the observation with the constant map for the localization on-line, is more preferred than SLAM. Lane markings are distinctive objects on road surfaces. From the mid-1980s, lane detection from camera sensors has received considerable attention. Those researches mainly focused on recognizing the lane markings on the road surface, but not localization [36,37,38,39,40]. Tao *et al.*, presented a localization method that built a map of the lane markings in a first stage, then the system conducted a map-matching process for improving the stand-alone GPS positioning error [41,42]. Schreiber *et al.*, developed a vision-based localization system using a stereo camera and a highly accurate map containing curbs and road markings [43]. Norman *et al.*, focused on the intersection scenario, and converted the map data to an image in a camera frame [44]. Nedevschi *et al.*, also developed an intersection localization system based on the alignment of visual landmarks perceived by an onboard stereo vision system, using the information from an extended digital map [29]. Christopher *et al.*, proposed to use a lane detection system for improving the localization performance when GPS suffers an outage [45]. Young *et al.*, developed an algorithm aimed at counting the sequence number of the occupied lanes based on multiple-lane detection, which were used for improving lateral localization [46]. In addition, the stop line detection function was employed to reduce the longitudinal error in localization as well [47,48]. All of these researches proposed to use the sensing technology to improve the positioning accuracy.

**Figure 1:**
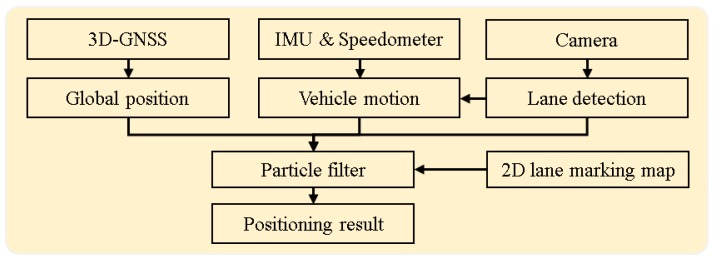
Flowchart of the proposed localization system.

Different with those related works, this paper focuses on the problem of vehicle self-localization in the most challenging environment, a city urban environment, and improves the positioning performance from two aspects: both GNSS positioning technology and sensor integration. The main contribution of this paper is to propose a multiple passive sensor-based localization system for the lane-level localization, which integrates the innovative 3D-GNSS positioning technique with an inertial sensor and onboard monocular camera. The flowchart of the proposed system is shown in Figure 1. In the integrated system, 3D-GNSS provides the global positioning result. The motion of vehicle is described via speedometer data from a Control Area Network bus, and heading direction from the Inertial Measurement Unit (IMU). The vision-based lane detection is firstly used for controlling the drift of the IMU. Moreover, the lane keeping and lane changing behaviors are obtained from the lane detection function. These behaviors describe the relative movement of the vehicle, which can further improve the lateral positioning error. Finally, we use a partite filter to integrate this information and the 2D lane marking map.

Our previous works [19,20,21] focused on the development and evaluation of 3D-GNSS for pedestrian applications. In addition, we only combined 3D-GNSS and inertial sensors for improving the localization performance in our previous works [27,28]. This paper further improves the localization performance by additionally integrating vision-based lane detection. This paper is an extension of the work presented in our conference paper [49]. Compared to the conference work, an idea of heading direction rectification is additionally described and evaluated, and more experimental results and discussion are presented in this paper as well.

We organize the remainder of the paper as follows: the 3D-GNSS positioning method and vision-based lane detection are presented in Section 2 and Section 3, respectively. The drift rectification method for an inertial sensor is described in Section 4. Section 5 explains the integration algorithm. The experimental results are demonstrated in Section 6. Finally, we will end the paper in Section 7 with conclusions and proposals of future work.

## 2. 3D-GNSS

**Figure 2:**
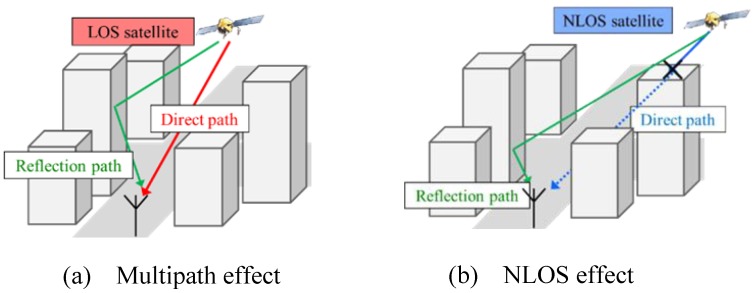
(**a**) Multipath effect [19]; (**b**) NLOS effect [19].

The integrated localization system considers the GNSS positioning result as the main information source. Generally speaking, GNSS positioning provides an estimation for localization, and the role of other sensors is to optimize the position around the GNSS result. A more accurate GNSS result makes it easier for the integrated system to achieve lane-level performance. Therefore, the performance of the GNSS positioning technique should be improved first. The satellite positioning techniques suffer from NLOS and multipath effects in urban canyons, as shown in Figure 2. This paper proposes to utilize 3D-GNSS to reduce both the multipath and NLOS effects. We briefly describe the idea of 3D-GNSS in this section, while the details of 3D-GNSS have been published in our previous works [19,20,21].

**Figure 3:**
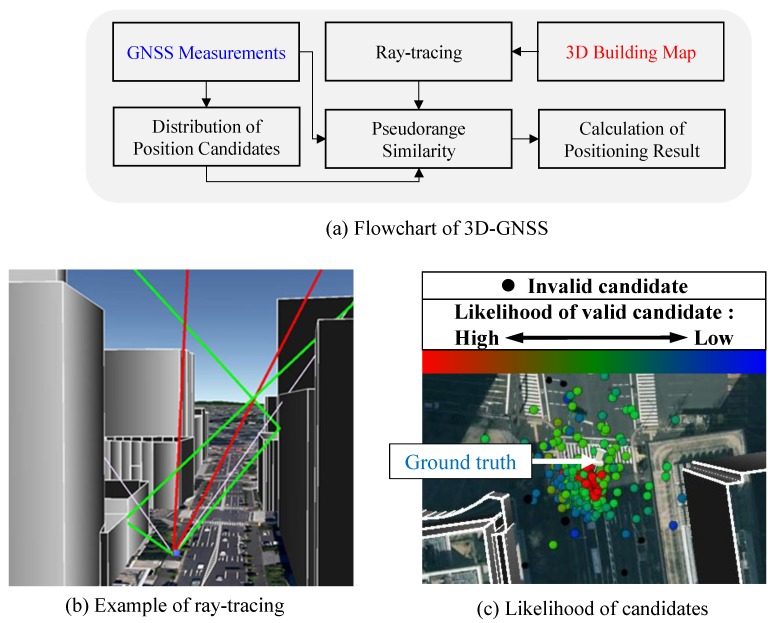
(**a**) Flowchart of 3D-GNSS; (**b**) Example of ray-tracing [19]; (**c**) Likelihood of candidates.

Figure 3a shows the flowchart of 3D-GNSS method. In order to analyze and correct the pseudorange error caused by multipath and NLOS, a 3D building map is needed. 3D-GNSS recognizes the reflected path of satellite signals using ray-tracing, and further estimates the reflection delay. Theoretically, the pseudorange simulation at the position of receiver should be the same as the pseudorange measurement. Thus, 3D-GNSS employs the candidate distribution algorithm to rectify the positioning error. Firstly, a set of position hypothesis, named candidates, are distributed around the previous positioning result and the position given by conventional GNSS receiver. Next, 3D-GNSS generates the pseudorange simulation from each candidate position using the ray tracing, as shown in Figure 3b.

There are three types of satellite conditions. The LOS signal is not affected by the buildings, and does not include the reflection delay. In the NLOS case, as shown in Figure 2b, the reflection delay is equal to the difference between the direct path and the reflection path. However, the third condition is more ambiguous, which is the multipath effect shown in Figure 2a. This research assumes that the signal in the multipath condition is about 6 dB weaker than the LOS signal, and the strobe correlator [50] with 0.2 chip spacing is adopted in the commercial receiver based on the experience. The research uses these principles to estimate the pseudorange delay in the multiple path condition. By using ray tracing, both the satellite condition and the reflection delay can be estimated.

The 3D-GNSS technique additionally checks the consistency of the satellite conditions by considering the signal strength. If the satellite conditions estimated using the signal strength conflict with the result from ray tracing, then the 3D-GNSS excludes the satellite from the following process. The probability of each position hypothesis is evaluated based on the similarity between the pseudorange measurement and the pseudorange simulation. Figure 3c indicates the different likelihood values of candidates using different colors. It is easy to see that the candidates around the ground truth position have higher weighting. The black dots are the invalid candidates, whose pseudorange similarity exceeds the defined threshold in this research of 10 m.

Finally, the 3D-GNSS provides the final rectified result by weighted averaging the positions of all the valid candidates. In this paper, we adopt the multiple satellite systems in 3D-GNSS, which includes not only GPS, but also GLObal NAvigation Satellite System (GLONASS) and Quasi Zenith Satellite System (QZSS).

## 3. Lane Detection from Monocular Camera

The lane detection method used in this research is highly inspired by Aly’s work [38]. However, different from the lane detection research described in [38], this research does not focus on the lane detection itself, but rather employs the vision-based lane detection to improve the accuracy of localization.

### 3.1. Inverse Perspective Mapping (IPM)

Before the lane detection, a top view of the road surface is generated, which is named Inverse Perspective Mapping (IPM). This has two advantages: the first one is that the perspective effect can be eliminated from the images, which makes the lanes become parallel [38]. The other one is that the road surface is represented in meters by the IPM. Thus, the prior information about the lane markings can be used easily.

**Figure 4:**
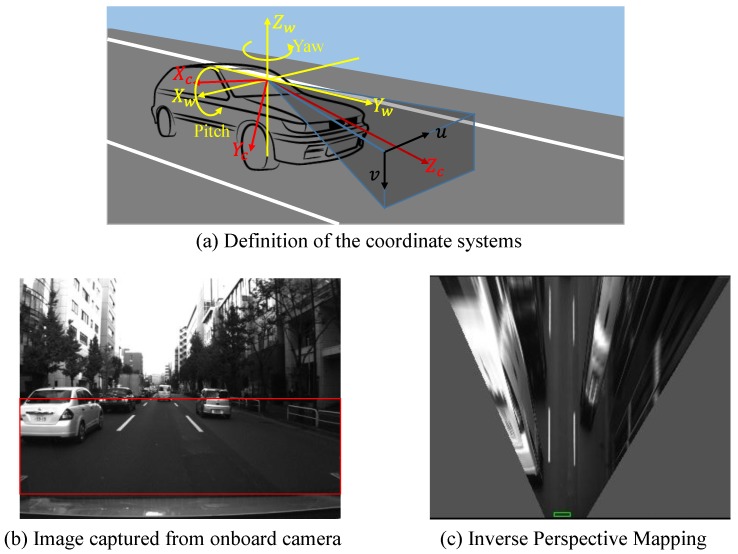
(**a**) Definition of the coordinate systems; (**b**) Original image from onboard camera; (**c**) IPM of the rectangle area in (**b**).

In order to generate the IPM, a flat road is assumed. In addition, for conducting this transformation, camera intrinsic parameters and camera extrinsic parameters are also used. As shown in Figure 4a, an image coordinate {*F_i_*} = {*u*, *v*}, a camera coordinate {*F_c_*} = {*X_c_*, *Y_c_*, *Z_c_*} and a world coordinate {*F_w_*} = {*X_w_*, *Y_w_*, *Z_w_*} are defined, respectively. The world coordinate is centered at the optical center of the camera, and *Z_w_* is perpendicular to the road surface. The camera coordinate *X_c_* axis is assumed to stay in the world coordinate *X_w_*-*Y_w_* plane. It means that the systems has a pitch angle *α* and yaw angle *β* for the optical axis, but the roll angle is zero. In addition, focal length of camera is (*f_u_*, *f_v_*), and optical center is (*c_u_*, *c_v_*). *h* is the height of the camera coordinate above the road plane. Thus, any point in the image coordinate can be projected to the road plane by the homogeneous transformation [38]:

(1) $$\left[\begin{array}{c} u\\ v\\ 1 \end{array}\right]=\left[\begin{array}{ccc} f_{u} & 0 & c_{u}\\ 0 & f_{v} & c_{v}\\ 0 & 0 & 1 \end{array}\right]\left[\begin{array}{c} x_{c}/z_{c}\\ y_{c}/z_{c}\\ 1 \end{array}\right]$$

(2) $$\left[\begin{array}{c} x_{c}\\ y_{c}\\ z_{c} \end{array}\right]=\left[\begin{array}{ccc} 1 & 0 & 0\\ 0 & \cos\left(\alpha +\frac{\pi }{2}\right) & -\sin\left(\alpha +\frac{\pi }{2}\right)\\ 0 & \sin\left(\alpha +\frac{\pi }{2}\right) & \cos\left(\alpha +\frac{\pi }{2}\right) \end{array}\right]\left[\begin{array}{ccc} \cos\beta & -\sin\beta & 0\\ \sin\beta & \cos\beta & 0\\ 0 & 0 & 1 \end{array}\right]\left[\begin{array}{c} x_{w}\\ y_{w}\\ -h \end{array}\right]$$

where Equation (1) represents the transformation from the camera coordinate to the image coordinate, and Equation (2) is the transformation from the world coordinate to the camera coordinate. Therefore, any point on the ground can be projected back to the image coordinate. Figure 4b shows an original image from onboard camera, and the region of interest (ROI) is marked by the red rectangle. Figure 4c visualizes the IPM image of the ROI, where the line boundaries of the lane become parallel.

### 3.2. Lane Detection

**Figure 5:**
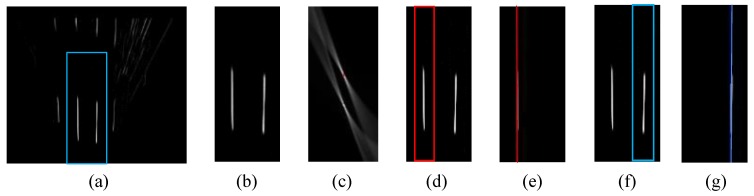
Lane detection [49], (**a**) Filtered IPM; (**b**) Lane detection ROI; (**c**) Hough transformation result of (**b**); (**d**) Rough area of the first line; (**e**) Result from RANSAC for the first line detection in (**d**); (**f**) Hypothesized area of the second line; (**g**) RANSAC result for the second line detection in (**f**).

For enhancing lane marking and reducing other disturbances, the IPM image is firstly smoothed by a Gaussian filter along the vertical direction, and then filtered by a second-derivative of Gaussian for the horizontal direction. The algorithm considers 2.5% pixels as the road marking and thresholds the filtered IPM [38], as shown in Figure 5a. In addition, the lane detection is limited in a rectangular area centered at the vehicle center. The width along the lateral direction is 7 m (two-lane width), and the farthest distance along the heading direction is 30 m. Figure 5b shows the lane detection ROI.

One lane consists of two line boundaries. Therefore, the lane detection problem is converted into a line detection one in the ROI. The line detection algorithm consistz of a Hough transform to locate the rough areas of lines, followed by a Random Sample Consensus (RANSAC) fitting to optimize the line detection results [38]. In fact, the two lines are represented by two local maxima in the Hough transform result, which is visualized in Figure 5c. Thus, the rough area of the first line is located by finding the global maximum in Hough transform space, as indicated by the red rectangle in Figure 5d. After that, the RANSAC line fitting is conducted in the red rectangle for detecting the first line. Figure 5e shows the result from RANSAC line fitting. In this paper, the lane width is assumed as 3.5 m. The hypothesized area of the second line can be determined based on this assumption. Then the RANSAC line fitting is used for the line detection again. Figure 5f,g demonstrate the hypothesized area of the second line and the result of the RANSAC line fitting, respectively. Thus, the position of the vehicle relative to the two line boundaries can be estimated.

### 3.3. Lane Keeping and Lane Changing Detection

**Figure 6:**
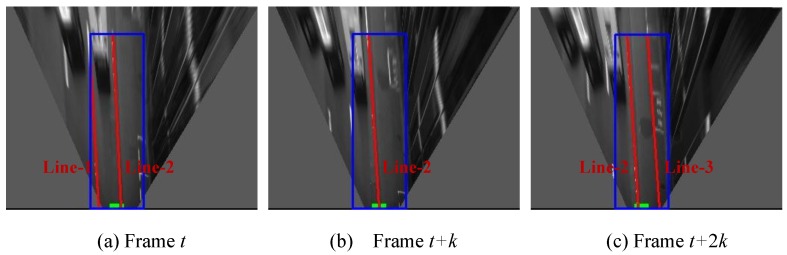
Detection of lane changing scenario; (**a**), (**b**) and (**c**) are line detection and tracking results at different frames. The blue rectangle is the ROI of lane detection, the red color lines are the detected lines. The attached number, e.g., “Line-1”, “Line-2” and “Line-3”, indicates the tracking results. The green rectangle shows the vehicle position.

One lane is represented by two line boundaries on the road surface. After the line boundaries are detected, we employ the particle filter to track each line. The purpose of the line tracking is to detect the lane keeping and changing behaviors. Figure 6 shows the line detection and tracking results in a lane changing process from frame *t* to *t* + 2*k*. In Figure 6, the blue rectangle is the ROI of lane detection, the red color lines are the detected lines. The attached number, e.g., “Line-1”, “Line-2” and “Line-3”, indicate the tracking results. The small green rectangle at the bottom of images denotes our vehicle position. From frame *t* to *t* + *k*, the Line-2 becomes close to the center of the vehicle, which is indicated in the Figure 6a,b. As time increases, the Line-2 gradually appears at the left side of the vehicle, as shown in Figure 6c. In this process, the position of the tracked Line-2 changes from the right side to the left side of the vehicle, which indicates the vehicle performs right-direction lane changing.

**Figure 7:**
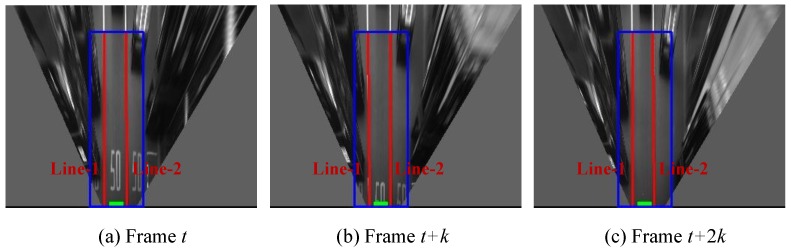
Detection of the lane keeping scenario; (**a**), (**b**) and (**c**) are the line detection and tracking results at different frames. The blue rectangle is the ROI of lane detection, the red color lines are the detected lines. The attached numbers, e.g., “Line-1” and “Line-2”, indicate the tracking results. The green rectangle shows the vehicle position.

Figure 7 shows the lane keeping scenario. The green rectangle denotes our vehicle position, which is located between Line-1 and Line-2 during the driving period. In Figure 7a–c, Line-1 always appears at the left and Line-2 always appears at the right side of the vehicle. Unlike the lane changing scenario, there is no line cross from one side to the other side of the vehicle in this period. Based on the relative position of the vehicle and the tracked lines, the lane keeping can be detected. This information will be used in the following integration.

## 4. Heading Direction Rectification for an Inertial Sensor

**Figure 8:**
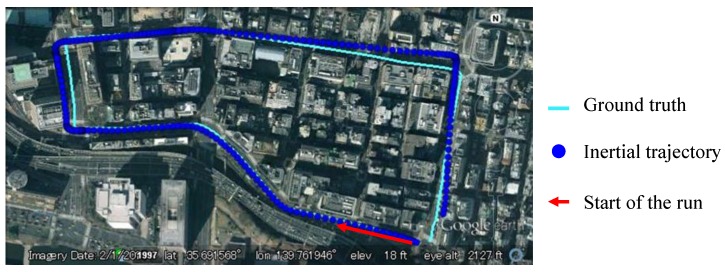
Drift effect of IMU sensor.

In this research, an Inertial Measurement Unit (IMU) sensor is employed to detect the heading direction of the vehicle. The IMU sensor outputs the acceleration, the angle rate and the value of angle along each axis. According to the IMU sensor specification, the pitch and roll angles are the difference from the body of the IMU sensor to the horizontal plane perpendicular to the gravity direction. In addition, the yaw angle is the accumulated angle changes since the sensor is reset or connected. Suppose that the heading direction has been calibrated relative to the true north direction, the vehicle velocity can be calculated by following equations:

(3) $$\left[\begin{array}{c} V_{north-east}\\ 0\\ V_{altitude} \end{array}\right]=\left[\begin{array}{ccc} \cos\omega & 0 & \sin\omega \\ 0 & 1 & 0\\ -\sin\omega & 0 & \cos\omega \end{array}\right]\left[\begin{array}{ccc} 1 & 0 & 0\\ 0 & \cos\lambda & -\sin\lambda \\ 0 & \sin\lambda & \cos\lambda \end{array}\right]\left[\begin{array}{c} V_{CAN}\\ 0\\ 0 \end{array}\right]$$

(4) $$\left[\begin{array}{c} V_{north}\\ V_{east}\\ V_{altitude} \end{array}\right]=\left[\begin{array}{ccc} \cos\theta & -\sin\theta & 0\\ \sin\theta & \cos\theta & 0\\ 0 & 0 & 1 \end{array}\right]\left[\begin{array}{c} V_{north-east}\\ 0\\ V_{altitude} \end{array}\right]$$

where, λ and ω are the roll and pitch angle of the IMU, respectively. θ is the heading direction from the true north direction. The vehicle velocity $V_{CAN}$ can be represented by $V_{north-east}$, and $V_{altitude}$ in Equation (3). The speed $V_{north-east}$ is further decomposed by Equation (4). Because we conducted the experiments in a flat area, the simplified North-East plane is adopted for the positioning. The GNSS positioning result at first epoch is defined as the original point of the North-East plane. The movement of the vehicle can be calculated by Equation (5):

(5) $$X_{k}=\left[\begin{array}{c} x_{north,k}\\ x_{east,k} \end{array}\right]=\left[\begin{array}{c} x_{north,k-1}\\ x_{east,k-1} \end{array}\right]+\Delta t\left[\begin{array}{c} V_{north,k-1}\\ V_{east,k-1} \end{array}\right]=\left[\begin{array}{c} x_{north,k-1}\\ x_{east,k-1} \end{array}\right]+\Delta t\left[\begin{array}{c} V_{north-east,k-1}\cdot \cos\theta_{k-1}\\ V_{north-east,k-1}\cdot \sin\theta_{k-1} \end{array}\right]$$

where, $\left(x_{north,k},x_{east,k}\right)$ denotes the vehicle position at time *k*. Figure 8 shows the trajectory generated from Equations (5). The trajectory is indicated by blue points and the ground truth is shown by cyan line. It is clear to see that the blue points are parallel with the ground truth at the beginning, but it is drifting with the increase of time.

This paper proposes to use the vision based lane detection to control the drift effect. When the lane keeping manner is detected, the vehicle heading direction is rectified as the direction of the occupied lane, which could be estimated from the 2D lane marking map. Otherwise, the heading direction is accumulated by the yaw angle rate provided by IMU sensor. This idea is formulized as follows:

(6) $$\theta_{k}=\{\begin{array}{c} \begin{array}{cc} \theta_{k}^{lane} & \text{,lane keeping} \end{array}\\ \begin{array}{cc} \theta_{k-1}+\Delta \theta_{k}^{IMU} & \text{,otherwise} \end{array} \end{array}$$

where, $\theta_{k}$ is the heading direction at time *k*, $\theta_{k}^{lane}$ is the lane direction obtained from 2D lane marking map. $\Delta \theta_{k}^{IMU}$ denotes the angle changing obtained from IMU sensor.

## 5. Integrated Localization of 3D-GNSS, Inertial Sensor and Lane Detection

In this research, multiple passive sensors are simultaneously adopted for positioning. 3D-GNSS, inertial sensor and lane detection are integrated in the particle filter. The particle filter is a nonparametric version of the Bayes filter, and has widely been applied. The particle filter represents a posterior using a set of particles ${\left\{x_{k}^{i}=(x_{k,north}^{i},x_{k,east}^{i})\right\}}_{i=1}^{n}$, $x_{k}^{i}$ is the two dimensional positioning, and *i* is the particle index, *k* is the time, *n* means the number of the particles. Each particle has an importance weight $w_{k}^{i}$. The vehicle position is recursively estimated by the following steps [51]:

1. Prediction: Create the particles ${\left\{x_{k}^{i},w_{k}^{i}\right\}}_{i=1}^{n}$ for time *k* based on the previous set of particles ${\left\{x_{k-1}^{i},w_{k-1}^{i}\right\}}_{i=1}^{n}$ and the control value $u_{k}$, based on the propagation model in Equation (5).
2. Correction: an weight of each particle in ${\left\{x_{k}^{i},w_{k}^{i}\right\}}_{i=1}^{n}$ is evaluated with the new observation $z_{k}$, according to certain observation model $w_{k}^{i}=p(z_{k}|x_{k}^{i})$. In this paper, $z_{k}$ includes two observation $G_{k}$ for 3D-GNSS positioning result and $V_{k}$ for lane detection result.
3. Resampling: the particle set ${\left\{x_{k}^{i},w_{k}^{i}\right\}}_{i=1}^{n}$ will be resampled based on the importance weight.

**Figure 9:**
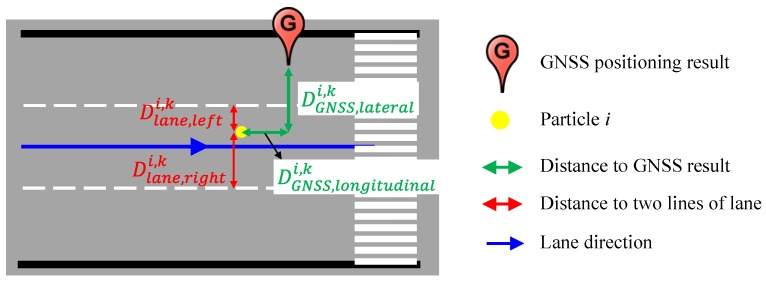
Distance estimation for a particle.

Figure 9 demonstrates the process of the weight evaluation for one particle. The yellow point is the particle *i*. The distance between the GNSS positioning result and the particle *i* is consist of the longitudinal distance $D_{GNSS,longitudinal}^{i,k}$ and the lateral distance $D_{GNSS,lateral}^{i,k}$ by referring to the direction of the occupied lane. Therefore, the probability computed thanks to 3D-GNSS measurement $p(G_{k}|x_{k}^{i})$ is represented as follows:

(7) $$p(G_{k}|x_{k}^{i})=\exp\left(\frac{-\left({\left(D_{GNSS,lateral}^{i,k}\right)}^{2}+{\left(D_{GNSS,longitudinal}^{i,k}\right)}^{2}\right)}{\sigma_{GNSS}^{2}}\right)$$

(8) $$p(G_{k}|x_{k}^{i})=p(G_{k,lateral}|x_{k}^{i})\cdot p(G_{k,longitudinal}|x_{k}^{i})$$

(9) $$p(G_{k,lateral}|x_{k}^{i})=\exp\left(\frac{-{\left(D_{GNSS,lateral}^{i,k}\right)}^{2}}{\sigma_{GNSS}^{2}}\right)$$

(10) $$p(G_{k,longitudinal}|x_{k}^{i})=\exp\left(\frac{-{\left(D_{GNSS,longitudinal}^{i,k}\right)}^{2}}{\sigma_{GNSS}^{2}}\right)$$

**Figure 10:**
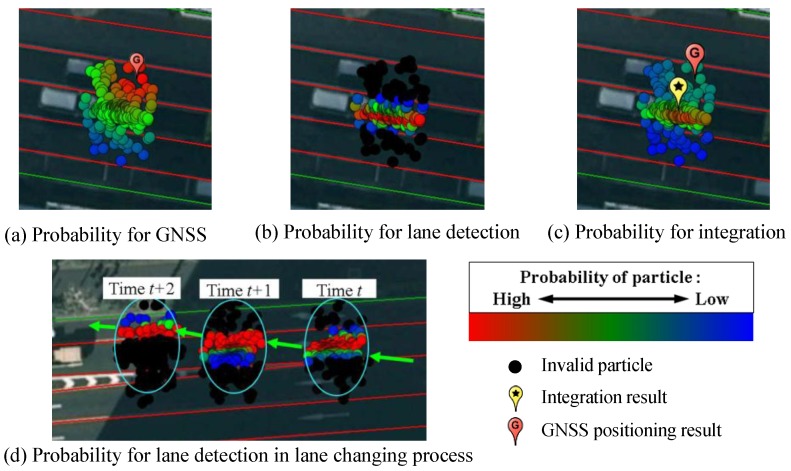
Particle probability evaluation.

This paper set the value of $\sigma_{GNSS}^{2}$ as nine square meters, which is tuned empirically. Figure 10a demonstrated the probability of the particles estimated from the GNSS measurement. The particles around of the GNSS position have high weighting.

This research optimizes the probability of the particles using the vision-based lane detection. The lane detection provides the distance from the vehicle center to right and left lines $\left(D_{right}^{k},D_{left}^{k}\right)$. In addition, the distance from each particle to right and left lines $\left(D_{lane,right}^{i,k},D_{lane,left}^{i,k}\right)$ can be estimated from the prepared 2D lane marking map, as shown in Figure 9. The probability computed thanks to lane detection measurement $p(V_{k}|x_{k}^{i})$ can be calculated as follows:

(11) $$p(V_{k}|x_{k}^{i})=\frac{1}{2}\left(p(V_{k,left}|x_{k}^{i})+p(V_{k,right}|x_{k}^{i})\right)$$

(12) $$p(V_{k,j}|x_{k}^{i})=\exp\left(\frac{-{\left(D_{lane,j}^{i,k}-D_{j}^{k}\right)}^{2}}{\sigma_{lane}^{2}}\right),\left(j\in \left\{right,left\right\}\right)$$

where, probability $p(V_{k,right}|x_{k}^{i})$ and $p(V_{k,left}|x_{k}^{i})$ correspond to the right line and left line, respectively. This paper empirically sets the variance $\sigma_{lane}^{2}$ as 0.25 m^2^. Figure 10b visualizes the probability $p(V_{k}|x_{k}^{i})$ estimated from the measurement of the lane detection. When the lane keeping is detected, the particles outside the occupied lane of the previous result, will be excluded in the calculation, and visualized as black dots in Figure 10b. The particles around the lane center have higher probability, because the vehicle runs along the lane center in experiments. It is important to note that the lane detection can sense the lateral position. It cannot perceive the position difference along the longitudinal direction. Therefore, we propose to integrate $p(V_{k}|x_{k}^{i})$ into $p(G_{k,lateral}|x_{k}^{i})$. Thus, the integrated probability is represented as:

(13) $$p(G_{k},V_{k}|x_{k}^{i})=\left(\left(1-\gamma )\cdot p(G_{k,lateral}|x_{k}^{i})+\gamma \cdot p(V_{k}|x_{k}^{i}\right)\right)\cdot p(G_{k,longitudinal}|x_{k}^{i})$$

where, γ is the importance weight of the lane detection measurement, which is set as 0.5 empirically. Figure 10c visualizes the integrated probability of all particles by different colors. Comparing to Figure 10a, the high weighting particles are not around of GNSS positioning result, but appear in the correct lane. $p(V_{k}|x_{k}^{i})$ in Equation (13) leads to this improvement. In addition, when the system detects lane changing, the operation for the particle exclusion follows the lane changing direction. Figure 10d demonstrates the valid particles corresponding to the lane detection measurement in the the lane changing case.

## 6. Experiments

**Figure 11:**
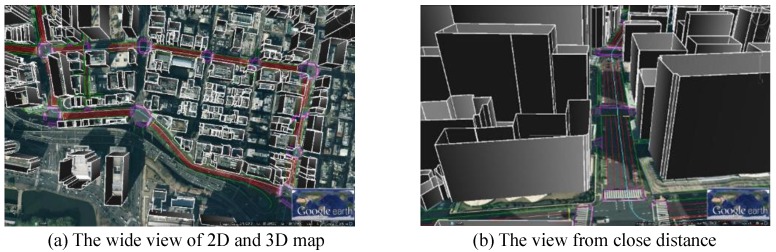
2D and 3D Maps used in this research.

We chose the Hitotsubashi area in Tokyo for experiments due to the tall building density. Figure 11 shows the developed 3D map for 3D-GNSS and 2D map for integration. We used two kinds of data to construct the 3D map. The first one is the 2-dimensional building footprint, which is provided by Japan Geospatial Information Authority. The other one is the Digital Surface Model (DSM) data, obtained from Aero Asahi Corporation. The DSM data includes the height information of the building [20]. The 2D map is generated from high resolution aerial images provided by NTT-geospace.

In the experiment, a u-blox EVK-M8 GNSS model, a commercial level receiver was adopted. It is produced by u-blox Company (Thalwil, Switzerland), and can receive signals from multiple-GNSS (GPS, GLONASS, and QZSS). We placed the u-blox receiver on the top of our vehicle to collect pseudorange measurements. In addition, an IMU sensor (AMU-3002A Lite, Silicon Sensing, Amagasaki-shi, Hyogo Prefecture, Japan) and speedometer recorder were used to measure the angle attitude and the vehicle velocity, respectively. Moreover, an onboard camera was installed in the vehicle, which captured the front view images when driving. These images are the input of the lane detection algorithm. In addition, we manually distinguished the ground truth trajectory of our vehicle from these images, and manually decided the occupied lane for the result evaluation. The driving distance is approximate 1500 m in each test.

**Figure 12:**
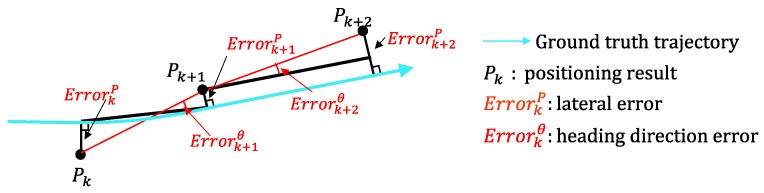
Definition of positioning error and heading direction error.

In the vehicle self-localization, it is more important to distinguish which lane the vehicle is in compared to the positioning accuracy. Therefore, both the lateral error and the correct lane rate are employed to estimate the performance of the localization system. Figure 12 shows the definition of the lateral error and the heading direction error. The lateral error $Error_{k}^{P}$ is the minimum distance from $P_{k}$ to the ground truth. The heading direction error $Error_{k}^{\theta }$ is the direction difference from the estimated trajectory to the ground truth trajectory. The heading direction error is used for the evaluation of the heading direction rectification.

### 6.1. Evaluation for Lane Detection

**Figure 13:**
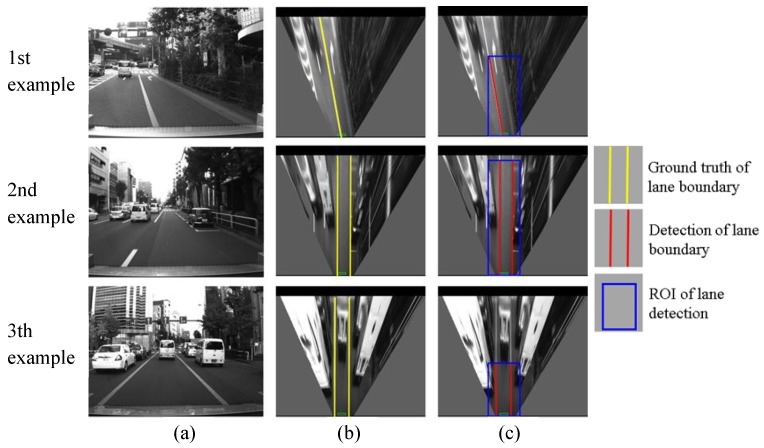
Examples of correct detections, (**a**) column shows images captured from camera; (**b**) column is IPM image and indicates the ground truth using yellow lines; (**c**) column shows lane detection result, where the blue rectangle is the ROI for lane detection and the red line is the detection result.

**Figure 14:**
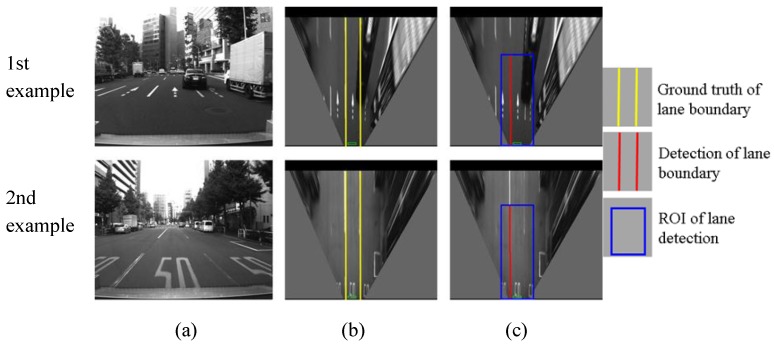
Examples of non-detection for one of lane boundaries, (**a**) column shows images captured from camera; (**b**) column is IPM image and indicates the ground truth using yellow line; (**c**) column shows lane detection result, where the blue rectangle is the ROI for lane detection and the red line is the detection result.

**Figure 15:**
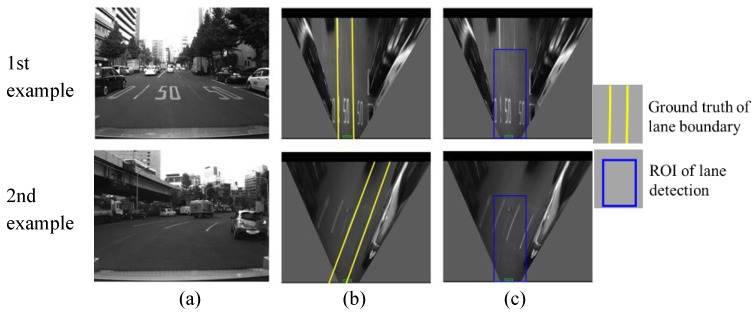
Examples of non-detection of both boundaries, (**a**) column shows images captured from camera; (**b**) column is IPM image and indicates the ground truth using yellow line; (**c**) column shows lane detection result, where the blue rectangle is the ROI for lane detection.

**Figure 16:**
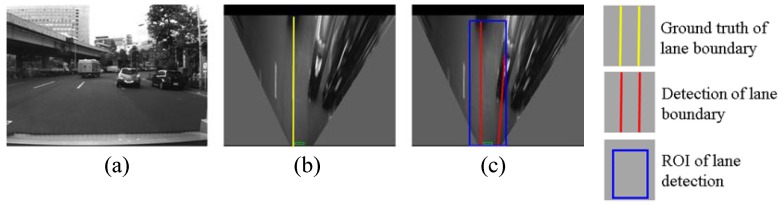
An example of false detection, (**a**) shows images captured from camera; (**b**) is IPM image and indicates the ground truth using yellow line; (**c**) shows lane detection result, where the blue rectangle is the region of interest (ROI) for lane detection and the red line is the detection result.

This section evaluates the lane detection by comparing the estimated lane with the hand-labeled ground truth. The cases of correction detection, partial non-detection, completed non-detection and false detection are shown in Figure 13, Figure 14, Figure 15 and Figure 16, respectively.

As shown in Figure 13, the developed lane detection can locate the lane boundary in different scenarios. The first row of Figure 13 shows a lane changing case, the crossed line is detected accurately. The second and the third rows demonstrate that there are many other vehicles around our vehicle in the experiments. Because the occupied lane is visible, the lane detection still works for finding the occupied lane. However, this kind of urban environment is challenging for detecting other lanes, because of the occlusions.

Figure 14 shows two examples of partial non-detection. In the two examples, the right lane boundaries are not detected and the left boundaries are correctly detected. The reasons are the occlusion of other vehicles in the first example, and the degradation of the lane markings in the second example. This case is not serious for the localization system. In the case of partial non-detection, the detected lane boundaries can still be used for integration. In the quantitative evaluation, the cases of Figure 13 and Figure 14 are considered as correct detections.

The complete non-detection means both left and right boundaries cannot be detected as shown in Figure 15. In the first example, the reason for the non-detection is the unclear lane marking on the road surface. In the second example, the lost detection of the left boundary is because the detection algorithm excludes the line, which does not cross the bottom of the ROI. The reason for the right boundary is that there are so few pixels of the line in the ROI. In the case of the completed non-detection, the localization system uses the GNSS positioning result for integration because of the lack of lane detection results. In addition, Figure 16 shows an example of false detection. A part of the right lane boundary is occluded by the front vehicle. The body of the vehicle presents a line feature, which causes the false detection.

The quantitative evaluation of the lane detection is illustrated in Table 1. The total number of frames including lanes is 5920. The developed lane detection algorithm can detect lanes in 95.3% of the frames. About 4.7% of the frames are complete non-detection ones, where the non-detection happens around curved road areas and on roads including unclear lane markings, such as Figure 15. In the detections, 99.6% of the frames are correct, such as the cases of Figure 13 and Figure 14. Only 0.4% are false detections, such as the case of Figure 16. Table 1 indicates that the lane detection displays high reliability.

**Table 1:** Quantitative evaluation of lane detection.

	Frames Including Lane (F)	Detection (D) (Figure 13, Figure 14 and Figure 16)	Non-Detection (ND) (Figure 15)	Correct Detection (CD) (Figure 13 and Figure 14)	False Detection (FD) (Figure 16)
**The number of frames**	5920	5642	278	5619	23
**Detection rate**	/	95.3% (D/F)	4.7% (ND/F)	99.6% (CD/D)	0.4% (FD/D)

### 6.2. Evaluation for Heading Direction Rectification

**Figure 17:**
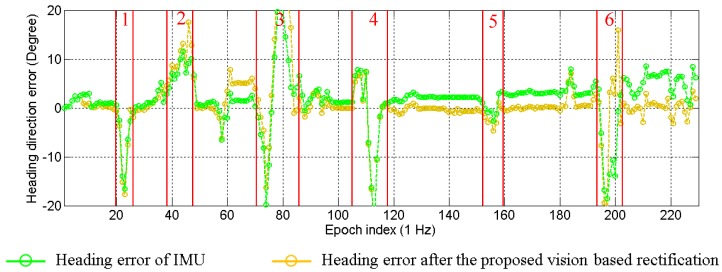
Effectiveness of the heading direction rectification.

**Figure 18:**
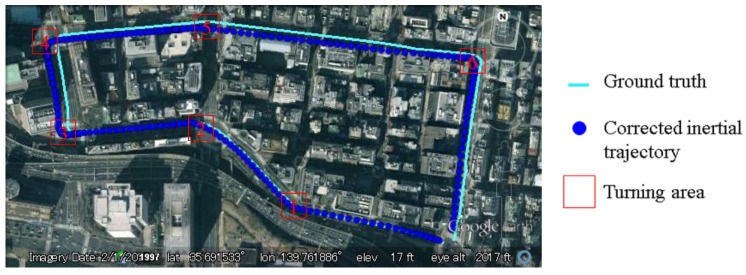
Inertial trajectory after heading rectification in the experiment of Figure 8.

As presented in Section 4, the IMU sensor suffers from the drift problem. The drift effect will generate errors in the propagation of particles. This paper proposes to rectify the heading direction, when lane keeping happens. Two types of heading direction errors in one experiment are plotted in Figure 17. The green line indicates the error of the IMU sensor, and the yellow line shows the error after the proposed rectification. The positive value means the difference in the counterclockwise direction. It is clear to see that the heading direction of the IMU sensor has about 5° drift by the end of the experiment. The error of the rectified heading direction changes around zero. This result proves that the vision-based lane detection can solve the drift problem. Actually, the experiment demonstrated in Figure 17 corresponds to the one discussed in Figure 8. In order to make the effectiveness of heading rectification more comprehensive, we plot the inertial trajectory using the rectified heading direction in Figure 18. After the rectification, the inertial trajectory is parallel to the ground truth trajectory. In Figure 17, the areas denoted as 1 to 6 are the turning areas, which are also indicated in Figure 18. The large error is caused by unconfident ground truth trajectory, because it is difficult to manually describe the accurate curve of the turning trajectory.

### 6.3. Evaluation for Localization

**Figure 19:**
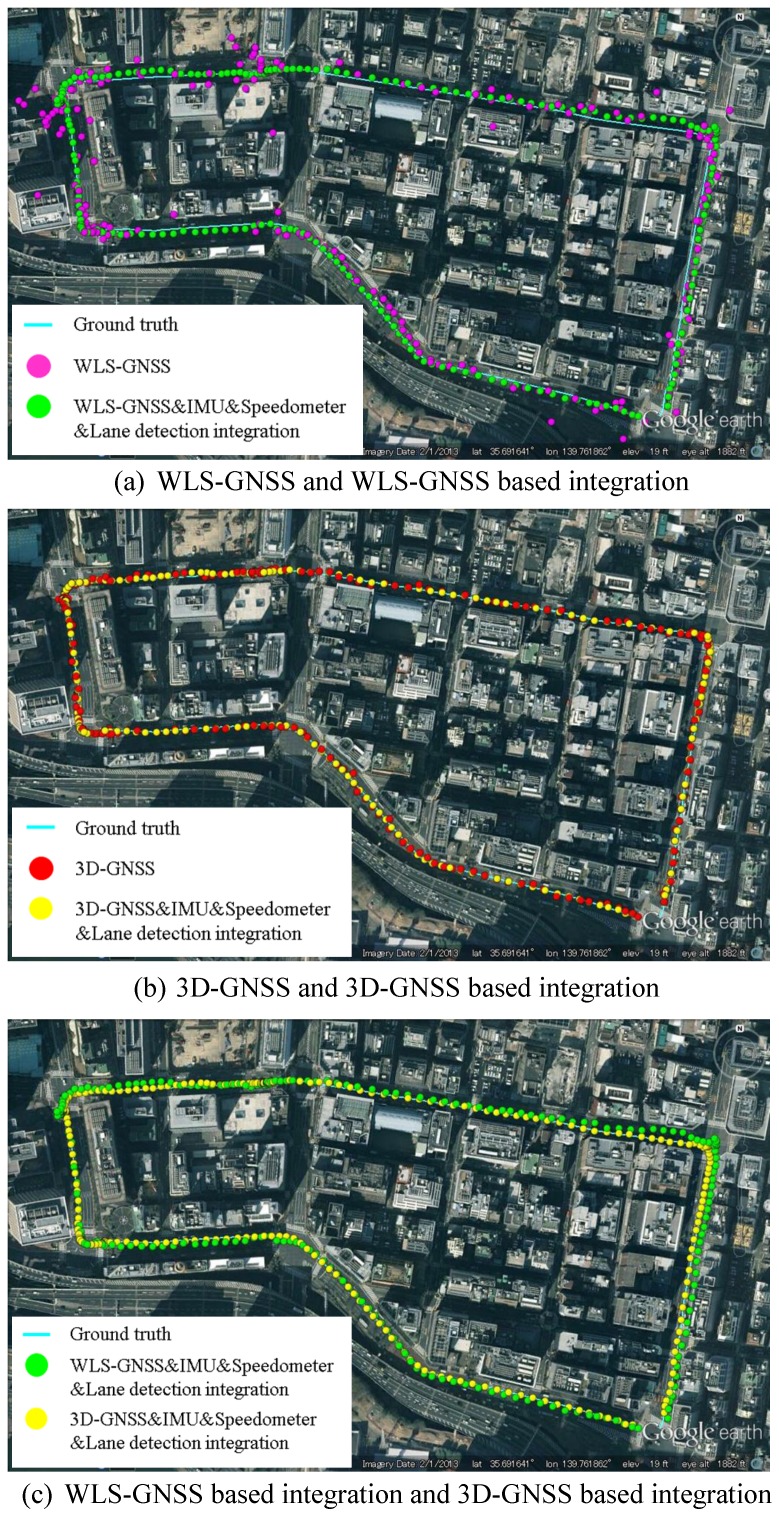
Positioning results of WLS-GNSS, 3D-GNSS and integration systems.

In this section, we focus on the evaluation of the positioning performance. In order to understand the benefit of the proposed 3D-GNSS in urban canyon environments, this paper compares the Weighted Least Square (WLS) GNSS-based integration and the proposed 3D-GNSS-based integration. Figure 19a shows the positioning results of WLS-GNSS and WLS-GNSS-based integration using purple dots and green dots, respectively. The 3D-GNSS and 3D-GNSS-based integration in the same test are shown in Figure 19b, using red dots and yellow dots. The ground truth is represented by the cyan line. In Figure 19a, the purple dots corresponding to the WLS-GNSS result are randomly spread over a wide area. On the contrary, the 3D-GNSS is much more accurate compared to the WLS-GNSS. Moreover, the 3D-GNSS-based integration also indicates better performance than WLS-GNSS-based integration, which can be understood by comparing the green dots and yellow dots in Figure 19c. Except for the GNSS positioning method, the two integration systems use the same algorithm. This proves that the more accurate the GNSS method is, the higher performance the integrated system has.

**Figure 20:**
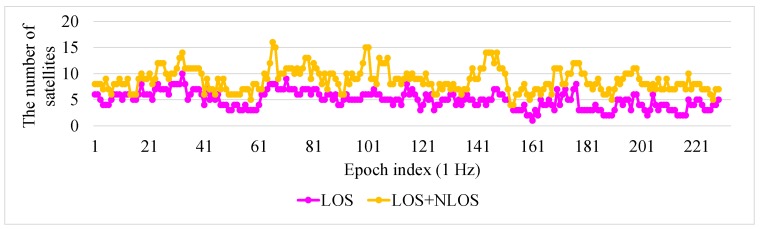
Condition of satellites in the experiment of Figure 19. Purple points indicate the number of LOS satellites from the ground truth position. Orange points are the number of satellites using 3D-GNSS (LOS + NLOS).

To explain the reason for the improvement in the 3D-GNSS method, the satellite conditions in this experiment are illustrated in Figure 20. In the experiment, the GNSS receiver can receive the signals from nine satellites on average. About five of the satellites are in LOS condition, and other satellites are NLOS. In the conventional WLS, the NLOS signal is used directly. Thus, the pseudorange error of the NLOS signal will lead to the positioning error. In the proposed 3D-GNSS positioning method, the 3D map information is adopted for distinguishing NLOS and multipath effects, and correcting the pseudorange errors. Therefore, the 3D-GNSS achieves better performance. It also provides an accurate basis for the integrated localization system.

We repeat three tests along the driving route. Table 2 shows the quantitative comparison based on the multiple tests. The comparison includes “WLS-GNSS”, “WLS-GNSS&IMU&Speedometer integration”, “WLS-GNSS&IMU&Speedometer&Lane detection integration”, “3D-GNSS”, “3D-GNSS&IMU&Speedometer integration”, and “3D-GNSS&IMU&Speedometer&Lane detection integration”. WLS-GNSS&IMU&Speedometer integration and 3D-GNSS&IMU&Speedometer integration exclude the lane detection function. As demonstrated in Table 2, the 3D-GNSS method also shows much better performance compared to WLS. About 63.2% of results are in the correct lane. Although 3D-GNSS cannot be directly used for vehicle self-localization, it has potential as the main source in the integration. After integrating with IMU and speedometer, the correct lane rate is increased and the mean error is improved to 1.2 m. Table 2 indicates the integration of GNSS, IMU, speedometer and lane detection has 93% correct lane rate and sub-meter accuracy.

**Table 2:** Comparison of different positioning methods.

Positioning Method	Positioning Error Mean (m)	Positioning Error Standard Deviation (m)	Correct Lane Recognition Rate
WLS-GNSS	7.53	10.06	12.9%
WLS-GNSS&IMU&Speedometer integration	5.72	5.34	16.1%
WLS-GNSS&IMU&Speedometer &Lane detection integration	3.16	2.56	37.4%
3D-GNSS	1.48	1.12	63.2%
3D-GNSS&IMU&Speedometer integration	1.17	0.84	79.1%
3D-GNSS&IMU&Speedometer&Lane detection integration	0.75	0.76	93.0%

**Figure 21:**
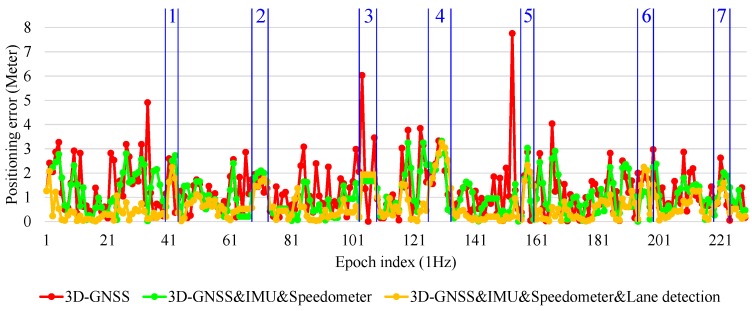
Positioning error of 3D-GNSS and 3D-GNSS-based integrations.

To demonstrate the performance of the 3D-GNSS based integration, Figure 21 shows the positioning error of the three types of localization methods in the experiment that has been discussed in Figure 19. Obviously, the 3D-GNSS&IMU&Speedometer&lane detection integration has the best performance. Most of the time, the positioning error is maintained under 1.5 m, but there are some areas where the positioning error is larger than 1.5 m. Those areas are indicated in Figure 21 and Figure 19b, correspondingly. The areas 1, 2, 3, 5, 6 and 7 in Figure 21 denote the intersections. In the intersections, the lane detection function does not work because of the lack of lane markings. Therefore, the positioning error cannot be reduced compared to 3D-GNSS&IMU&Speedometer integration. This result indicates that it is necessary to develop other road marking recognition functions for intersection areas, which is expected to improve the positioning error in those intersection areas.

**Figure 22:**
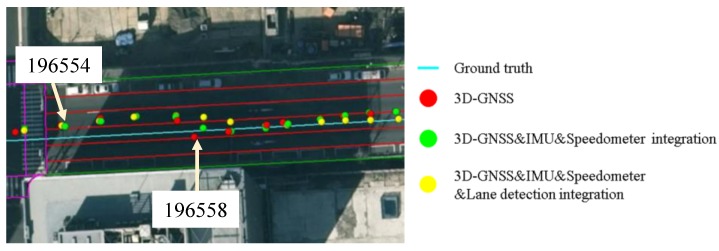
Demonstration of the effect of the 3D-GNSS in the proposed integration.

Area 4 is in the road link. This area is enlarged and shown in Figure 22. The vehicle enters this road link from epoch 196554. From the epoch 196554 to epoch 196557, 3D-GNSS results are in the incorrect lane (the left lane of the ground truth). At the same time, the camera detects lane keeping behavior. Therefore, the integrated localization system gives an incorrect positioning result, as indicated by the yellow dots. From epoch 196558, the 3D-GNSS appears in the correct lane, and the result of the integration gradually becomes correct because of the effect of the 3D-GNSS. The epochs from 196554 to 196557 correspond to the area 4 in Figure 21 and Figure 19b. This case study explains the reason for the 7% failure in the occupied lane recognition. It is important to note that this case also demonstrates that the positioning result can be corrected from an incorrect lane assignment by the 3D-GNSS after several epochs.

**Figure 23:**
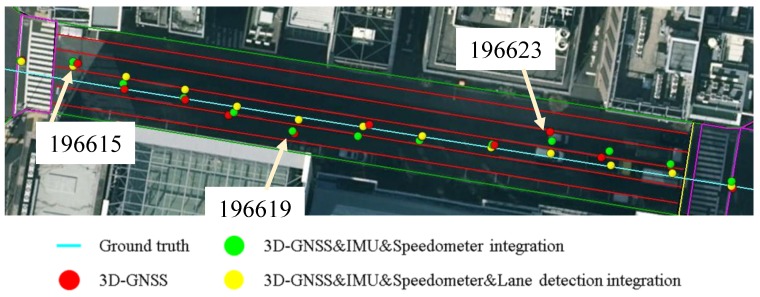
Demonstration of the effect of the lane detection in the proposed integration.

Figure 23 shows another case, which can demonstrate the benefit of the lane detection. At the epoch 196619 and epoch 196623, the 3D-GNSS&IMU&Speedometer integration (green dots) appears in the incorrect lane because of the 3D-GNSS error. The lane detection increases the probability of the particles being in the correct lane, which is explained in Figure 10. The lane detection reduces the lateral positioning error by correctly adjusting the probability of particles, which is the reason for the improvements indicated in Table 2 and Figure 21.

## 7. Conclusions

This research extended our previous 3D-GNSS work for vehicle positioning in city environments. As indicated in the experimental results, the innovative 3D-GNSS positioning technique achieved 1.5 m mean positioning error and a 63% lane recognition rate by reducing the multipath and NLOS effects. However, it is still difficult to satisfy the sub-meter requirement by only using 3D-GNSS. This paper proposed the integration of multiple on-board sensors, 3D-GNSS, inertial sensors and an on-board monocular camera for improving the accuracy of vehicle positioning. In the integration system, a lane detection algorithm is developed, in order to recognize the lane keeping/changing behavior. Finally, the particle filter integrates the three sources, 3D-GNSS, vehicle motion and lane detection for localization. In the integration, the drift problem of the inertial sensor is effectively controlled by using lane detection. Moreover, the lane detection function provides an additional measurement to optimize the positioning result along lateral direction. The experiments conducted in the urban traffic environment have demonstrated that the proposed system achieved a 93% correct lane recognition rate and sub-meter accuracy. In the near future, the sensing technology for the intersection scenario will be considered to improve the localization accuracy. In addition, more difficult scenarios will be discussed, such as under bridgea or in tunnels, where the GNSS signal is in outage conditions.
